# Mexican species of the genus *Stethantyx* Townes (Hymenoptera, Ichneumonidae, Tersilochinae)

**DOI:** 10.3897/zookeys.360.6362

**Published:** 2013-12-06

**Authors:** Andrey I. Khalaim, Enrique Ruíz-Cancino

**Affiliations:** 1 Facultad de Ingeniería y Ciencias, Universidad Autónoma de Tamaulipas, Cd. Victoria 87149, México; 2 Zoological Institute, Russian Academy of Sciences, Universitetskaya nab. 1, St. Petersburg 199034, Russia

**Keywords:** Key, Neotropical region, new records, new species, North America, taxonomy

## Abstract

Six species of the genus *Stethantyx* Townes are found to occur in Mexico. One species, *S. mexicana* **sp. n.**, is described as new, and four recently described Neotropical species, *S. alajuela* Khalaim & Broad, *S. heredia* Khalaim & Broad, *S. osa* Khalaim & Broad and *S. sanjosea* Khalaim & Broad, are new records from Mexico. A key to species of *Stethantyx* occurring in Mexico is provided.

## Abstract (es)

En México se han encontrado seis especies del género *Stethantyx* Townes. Se describe la especie *S. mexicana* **sp. n.** Cuatro especies descritas recientemente para la región Neotropical son nuevos registros para México: *S. alajuela* Khalaim & Broad, *S. heredia* Khalaim & Broad, *S. osa* Khalaim & Broad y *S. sanjosea* Khalaim & Broad. Se elaboró la clave de especies de *Stethantyx* que ocurren en México.

## Introduction

*Stethantyx* is a large predominantly Neotropical genus with 42 described and many undescribed species (Yu et al. 2012; Khalaim and Broad 2013; Khalaim et al. 2013). Along with the Neotropical monotypic genus *Megalochus* Khalaim & Broad, it forms the *Stethantyx* genus-group, characterized by the fore wing with abscissae of the radius meeting at an obtuse angle (more than 90°), intercubitus and abscissa of cubitus between intercubitus and second recurrent vein not or very weakly thickened, hind wing with nervellus more or less vertical, and prepectal carina with upper end not reaching anterior margin of mesopleuron, continuing above and backwards to the subtegular ridge (Khalaim and Broad 2013). *Stethantyx* differs from *Megalochus* by the longer antennae, first metasomal segment stouter and trapeziform in cross-section, presence of glymmae, and smooth or granulate (not rugulose) propodeum and metapleuron.

Only two native species, *Stethantyx crassa* Horstmann and *Stethantyx nearctica* Townes, and *Stethantyx parkeri* (Blanchard), introduced from South America, are known to occur in the Nearctic region (Horstmann 2010). In the Neotropical region, 22 species were recently described from Costa Rica (Khalaim and Broad 2013); in South America, eight species (including *Stethantyx parkeri*) have been described from Argentina, Brazil and Uruguay (Blanchard 1945, Graf 1980), and 16 species (including ten new species), have been found to occur in Western Amazonia (Khalaim et al. 2013). Two unidentified species of *Stethantyx* were also reported from Cuba (Fernández-Triana et al. 2006).

Some species of *Stethantyx* were reared from the beetle families Nitidulidae and Curculionidae (Coleoptera). In the Nearctic region, *Stethantyx crassa* was reared from *Cryptarcha* sp. and/or *Lobiopa undulata* Say (Nitidulidae) from sap spots on oak (Williams et al. 1984, Horstmann 2010), and *Stethantyx nearctica* was reared from *Balaninus* sp. (Curculionidae) on Quercus alba L. (Fagaceae), probably from acorns (Horstmann 2010). In South America, three species, *Stethantyx argentiensis* (Blanchard), *Stethantyx parkeri* and one unidentified species, all parasitizing vegetable weevils (Curculionidae: *Listroderes* spp.) (Parker et al. 1950), were introduced to the southern U.S.A. and eastern Australia for the control of *Listroderes obliquus* Klug, a pest of many cultivated vegetables (Kerrich 1961, Wilson and Wearne 1962, Clancy 1969, Gauld 1984).

Only one species, *Stethantyx nearctica*, was previously known from northern Mexico (Horstmann 2010). In this paper we review six Mexican species of *Stethantyx*, including one new species and four new records. A key for identification of these species is given.

## Methods

The ichneumonid collection of the Universidad Autónoma de Tamaulipas, Cd. Victoria, Mexico (further UAT) was studied. One specimen of *Stethantyx* was borrowed from the Essig Museum of Entomology, University of California, Berkeley, U.S.A. (further EMEC). Some specimens were deposited in the Zoological Institute of the Russian Academy of Sciences, St. Petersburg, Russia (further ZISP).

Morphological terminology predominantly follows Townes (1969, 1971) with changes according to Khalaim (2011). Photographs were taken at ZISP with a DFC 290 digital camera attached to a Leica MZ16 stereomicroscope; the partialy focused photographs were assembled with Helicon Focus software. Photographs of wings of *Stethantyx mexicana* sp. n. (Fig. 16) were taken from a microscope slide prepared with Canada balsam.

## Results

### Stethantyx

Townes, 1971

http://species-id.net/wiki/Stethantyx

#### Type species.

*Stethantyx nearctica* Townes, 1971.

#### Key to species of *Stethantyx* occurring in Mexico

**Table:** 

1	Mesosoma entirely black (Figs 20, 23). Ovipositor sheath twice as long as first tergite	*Stethantyx nearctica* Townes
–	Mesosoma entirely or predominantly reddish orange (Figs 8, 17, 25). Ovipositor sheath 1.2–1.8 times as long as first tergite (unknown for *Stethantyx mexicana* sp. n.)	2
2	Head reddish orange, frons sometimes infuscate centrally (Figs 28, 29). Propodeum with narrow basal area (Fig. 30)	*Stethantyx sanjosea* Khalaim & Broad
–	Head black, face and clypeus sometimes reddish brown or yellow (Figs 2, 6, 7, 10, 11, 24, 25). Propodeum not as above: with basal keel (Figs 9, 18), broad basal area (Fig. 26) or longitudinal wrinkles mediodorsally (Fig. 5)	3
3	Flagellum with conspicuous pale band (Fig. 1). Face yellow (Fig. 2), with strong prominence centrally. Notaulus strongly impressed, distant from anterolateral margin of mesoscutum (Fig. 3). Propodeum usually without distinct basal area, with longitudinal wrinkles dorsally (Fig. 5)	*Stethantyx alajuela* Khalaim & Broad
–	Flagellum black, without pale band (as in Fig. 27). Face yellow or black, with weak prominence centrally. Notaulus not impressed, as wrinkle or small tubercle distant from anterolateral margin of mesoscutum (Figs 6, 10), or completely absent. Propodeum with distinct basal area (Fig. 26) or basal keel (Figs 9, 18)	4
4	Propodeum with broad basal area (Fig. 26). Propodeal spiracle separated from pleural carina by 1.5–2.0 times diameter of spiracle (Fig. 25). Face black (Fig. 24)	*Stethantyx osa* Khalaim & Broad
–	Propodeum with basal keel (Figs 9, 18). Distance between propodeal spiracle and pleural carina less than one diameter of spiracle (Figs 8, 17). Face usually reddish brown or yellowish (Figs 7, 11)	5
5	Mesoscutum, scutellum and propodeum black, strongly contrasting with remaining mesosoma (Fig. 17)	*Stethantyx mexicana* sp. n.
–	Mesosoma uniformly reddish orange (Fig. 8)	*Stethantyx heredia* Khalaim & Broad

### Stethantyx alajuela

Khalaim & Broad, 2013

http://species-id.net/wiki/Stethantyx_alajuela

Figs 1–5

#### Material examined.

**Mexico:** Veracruz, Estación Biológica Los Tuxtlas, 480 m, 2.VI.1986, coll. P. Sinaca, 1 female (UAT).

#### Distribution.

Mexico (Veracruz), Costa Rica, Ecuador, Peru, Paraguay. First record from Mexico.

**Figures 1–5.**
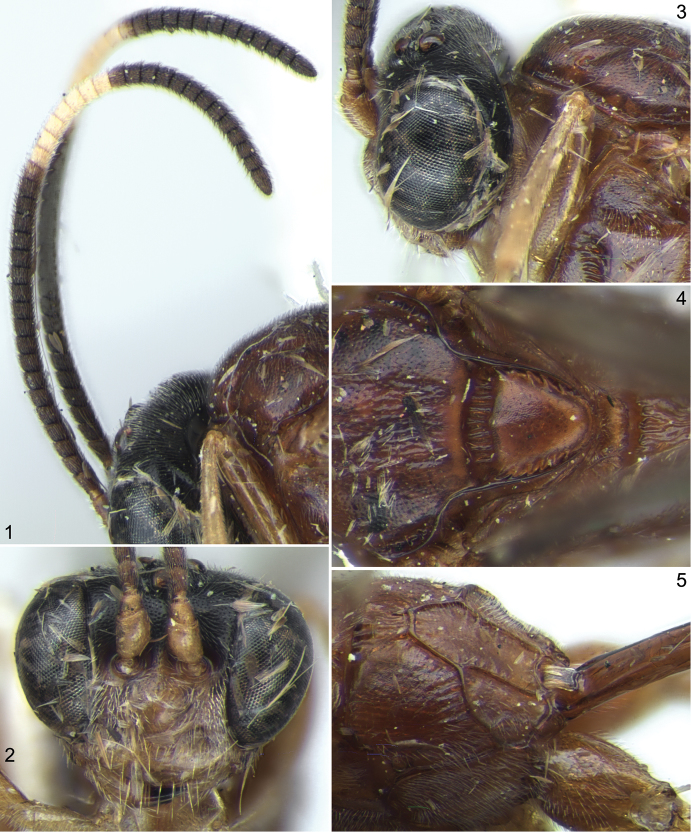
*Stethantyx alajuela* Khalaim & Broad, ♀: **1** antennae, lateral view **2** head, frontal view **3** head and anterior part of mesosoma, lateral view **4** mesoscutum and scutellum, dorsal view **5** propodeum, dorsolateral view.

### Stethantyx heredia

Khalaim & Broad, 2013

http://species-id.net/wiki/Stethantyx_heredia

Figs 6–9

#### Material examined.

**Mexico:** Chiapas, Jaltenango, Reserva El Triunfo, Malaise trap, 19–22.VII.1997, coll. A. González Hernández, 1 female (UAT). Quintana Roo, Rancho #3, Valle Hermoso, 21.VII.1993, coll. H. Delein, 2 females (UAT).

#### Distribution.

Mexico (Chiapas, Quintana Roo), Costa Rica, Ecuador, Peru. First record from Mexico.

**Figures 6–9.**
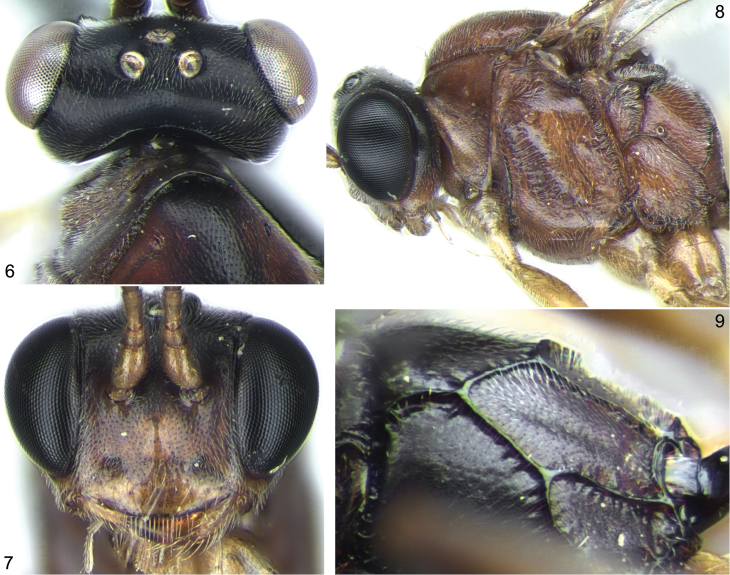
*Stethantyx heredia* Khalaim & Broad, ♀: **6** head and anterior part of mesosoma, dorsal view **7** head, frontal view **8** head and mesosoma, lateral view **9** propodeum, dorsolateral view.

### Stethantyx mexicana

Khalaim & Ruíz-Cancino sp. n.

http://zoobank.org/8FD563AE-170A-4D0E-A09E-F38CD0B5B343

http://species-id.net/wiki/Stethantyx_mexicana

Figs 10 –19

#### Comparison.

Differs from its North and Central American congeners by the combination of brownish orange mesosoma with black mesoscutum, scutellum and propodeum (Fig. 17), and propodeum with basal keel (Fig. 18). In the key to Costa Rican species of *Stethantyx* (Khalaim and Broad 2013) it runs to couplet 7 but differs in this couplet from both species, *Stethantyx tenoriosa* Khalaim & Broad and *Stethantyx mesoscutator* Khalaim & Broad, by the propodeum with basal keel (basal area in two other species), and from *Stethantyx mesoscutator* also by the black propodeum. The new species is morphologically similar to *Stethantyx heredia* as both have propodeum with basal keel but differs (besides colour pattern of mesosoma) by the somewhat longer temple and longer second metasomal tergite. *Stethantyx mexicana* sp. n. is a very distinct species, and despite the fact that it is described only from males, it may easily be distinguished from all known species of *Stethantyx*.

#### Description.

Male. Body length 4.8 mm. Fore wing length 3.45 mm.

**Figures 10–15.**
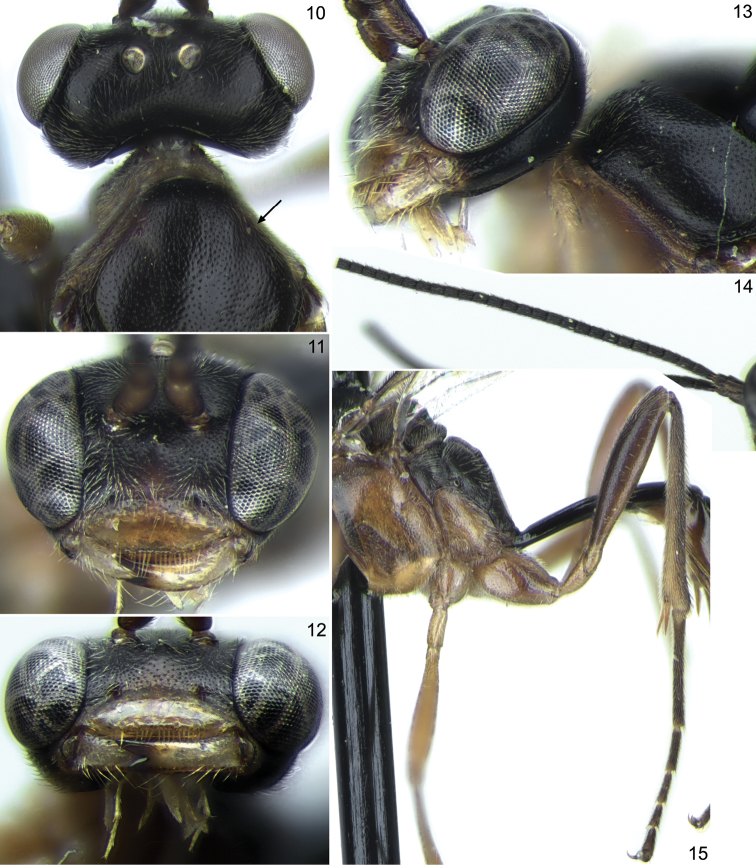
*Stethantyx mexicana* sp. n., ♀, holotype (except Figs **10** and **12**): **10** head and anterior part of mesosoma, dorsal view **11** head, frontal view **12** head, ventral view **13** head and anterior part of mesosoma, lateral view **14** antenna (apex absent), lateral view **15** posterior part of mesosoma and hind leg, lateral view.

**Head:** Roundly narrowed behind eyes in dorsal view (Fig. 10); temple almost 0.6 times as long as eye width. Mandible with upper tooth much longer than lower tooth (Fig. 12). Clypeus lenticular, flat in lateral view, about 2.5 times as broad as long, smooth, punctate on upper 0.3 (Fig. 11). Malar space 0.2 times as long as basal width of mandible (Fig. 13). Flagellum of antenna with over 28 segments (tips of all antennae absent), distinctly tapered towards apex; all flagellomeres, except the basal one, 1.3–1.4 times as long as broad (Fig. 14); flagellomeres 4 to 14 bear finger-shaped subapical structures on outer surface. Face and frons finely and densely punctate on finely granulate dull background. Vertex finely and densely punctate (punctures distinct medially and indistinct laterally) on very finely granulate, weakly shining background. Temple with very fine, mostly indistinct punctures, weakly shining. Face with weak prominence centrally. Occipital carina complete.

**Mesosoma:** Notaulus as small tubercle (in both paratypes) or short wrinkle (in holotype) distant from anterolateral margin of mesoscutum (Figs 10, 13). Mesoscutum very finely (sometimes indistinctly) punctate on very finely granulate, dull background. Scutellum with lateral longitudinal carinae extending from its base to posterior 0.6–0.7. Mesopleuron finely but distinctly punctate on smooth and shining background (area just above foveate groove impunctate), peripherally finely granulate. Foveate groove situated more or less in centre of mesopleuron, strongly oblique, deep and crenulate (Fig. 17). Propodeum with all carinae strong, transverse carina without adjacent wrinkles (Fig. 18). Dorsolateral area very shallowly granulate, dull, with sparse indistinct punctures. Basal keel of propodeum almost 0.4 times as long as apical area (Fig. 18). Propodeal spiracle big, adjacent to pleural carina (Fig. 17). Apical area flat, pointed anteriorly (Fig. 18).

**Figures 16–19.**
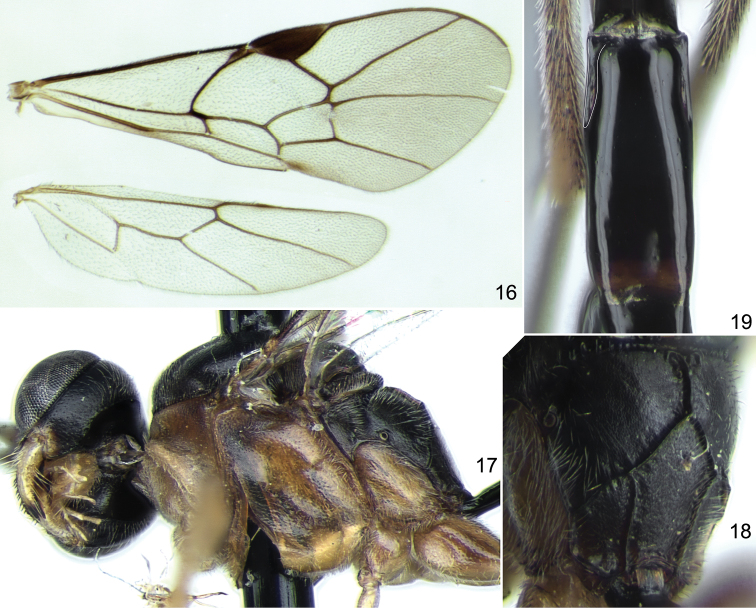
*Stethantyx mexicana* sp. n., ♀, holotype (except Fig. **16**): **16** wings **17** head and mesosoma, lateral view **18** propodeum, dorsolateral view **19** second tergite, dorsal view.

**Wings:** Fore wing (Fig. 16) with first and second sections of radius angled about 130°. Intercubitus 1.5–2.0 times as long as abscissa of cubitus between intercubitus and second recurrent vein. Metacarp almost reaching apex of fore wing. Hind wing (Fig. 16) with nervellus distinctly inclivous.

**Legs:** Slender (Fig. 15). Hind femur slightly clavate, 4.7 times as long as broad and 0.8 times as long as tibia. Hind spurs slightly curved at apex. Tarsal claws rather long, not pectinate.

**Metasoma:** First tergite slender, 4.6 times as long as posteriorly broad, entirely smooth. Glymma situated somewhat behind centre of tergite, moderately large, groove between glymma and ventral part of postpetiole weak but distinct in two paratypes and absent in the holotype. Second tergite almost 2.6 times as long as anteriorly broad (Fig. 19). Thyridial depression very long, more than 5.0 times as long as broad (Fig. 19).

**Coloration:** Head black with lower part of face slightly reddish brown, clypeus and malar space yellow. Palpi and mandible (teeth reddish black) yellow. Scape and pedicel of antenna dark brown or brownish black; flagellum black, probably without pale band. Mesosoma brownish orange; propleuron and lower part of pronotum yellowish; mesoscutum, scutellum and propodeum dark brown or black, strongly contrasting with remaining mesosoma. Tegula fuscous. Pterostigma dark brown. Legs yellow to brownish yellow; hind leg with coxa with brown mark on extero-outer surface, femur brown to dark brown, and tibia and tarsus blackish. First tergite black. Metasoma behind first tergite predominantly dark brown (almost black dorsally), ventrally yellowish, second and following tergites with pale band posteriorly.

Female unknown.

#### Variability.

The three specimens are very uniform, with minor variation in structure and coloration. One paratype has the basal keel of the propodeum centrally indistinct.

#### Etymology.

From the type locality, Mexico.

#### Material examined.

Holotype male, Mexico, Chiapas, Reserva El Triunfo, Jaltenango, Red de golpeo, 15°39'22"N, 92°48'31"E, 1400 m, 21.VII.1997, coll. A. González Hernández, CIB 97-063a (UAT).

#### Paratypes.

Mexico, Chiapas, Palo Gordo, screen sweeping, 21.VI.1997, coll. A. González Hernández, 2 males (UAT, ZISP).

#### Distribution.

Mexico (Chiapas).

### Stethantyx nearctica

Townes, 1971

http://species-id.net/wiki/Stethantyx_nearctica

Figs 20–23

#### Material examined.

**Mexico:** Tamaulipas, Cd. Victoria, 20.X.2007, coll. E. Ruíz-Cancino, 1 female (UAT). **U.S.A.:** Florida, Jefferson Co., Monticella, University of Florida, Malaise trap, 5–19.I, 5.X–30.XI.2001, coll. R. Mitzel, 2 females (UAT, ZISP).

#### Distribution.

U.S.A. (Arizona, District of Columbia, Florida, Iowa, Maryland, North Carolina, South Carolina, Texas, Virginia), Mexico (Nuevo León, Tamaulipas).

#### Biology.

Reared in Virginia, U.S.A., from *Balaninus* sp. (Curculionidae) on *Quercus alba* L. (Fagaceae), probably from acorns (Horstmann 2010).

**Figures 20–25.**
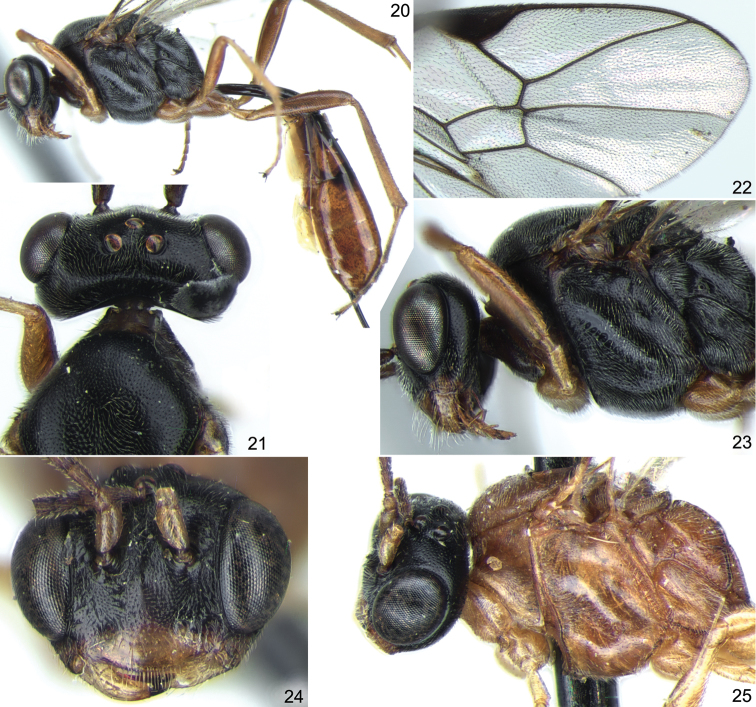
*Stethantyx nearctica* Townes, ♀: **20** habitus (without antennae and wings), lateral view **21** head and anterior part of mesosoma, dorsal view **22** apex of fore wing **23** head and mesosoma, lateral view. *Stethantyx osa* Khalaim & Broad, ♀: **24** head, frontal view **25** head and mesosoma, lateral view.

### Stethantyx osa

Khalaim & Broad, 2013

http://species-id.net/wiki/Stethantyx_osa

Figs 24 –26

#### Material examined.

**Mexico:** Nayarit, Jesus Maria, 27.VII.1955, coll. B. Malkin, 1 female (EMEC).

#### Distribution.

Mexico (Nayarit), Costa Rica. First record from Mexico.

**Figures 26–31.**
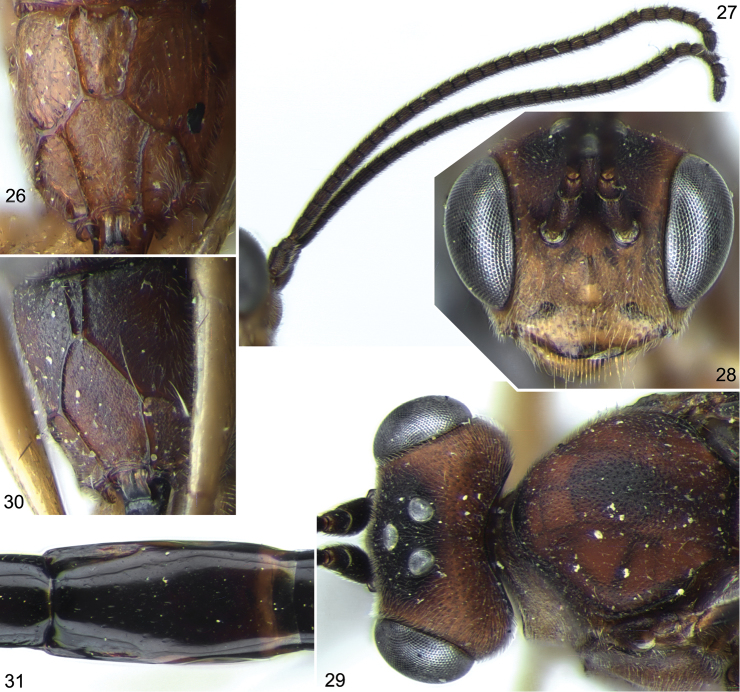
*Stethantyx osa* Khalaim & Broad, ♀: **26** propodeum, dorsal view. *Stethantyx sanjosea* Khalaim & Broad, ♀: **27** antennae, lateral view **28** head, frontal view **29** head and anterior part of mesosoma, dorsal view **30** propodeum, dorsal view **31** second tergite, dorsal view.

### Stethantyx sanjosea

Khalaim & Broad, 2013

http://species-id.net/wiki/Stethantyx_sanjosea

Figs 27–31

#### Material examined.

**Mexico:** Tamaulipas, Gómez Farías, Canindo, 1400 m, Malaise trap, 28–30.VII.1993, coll. J.B. Woolley, 3 females (UAT). Chiapas, Jaltenango, Reserva El Triunfo, Malaise trap, 19–22.vii.1997, coll. A. González Hernández, 1 female (UAT).

#### Distribution.

Mexico (Tamaulipas, Chiapas), Costa Rica, Ecuador, Peru. First record from Mexico.

## Discussion

Four species of *Stethantyx*, previously known from Costa Rica and South America, were recorded mostly from central and southern parts of Mexico and, along with the new species, belong to the Neotropical complex of species. Only one species, *Stethantyx nearctica*, occurring in northern Mexico and U.S.A., belongs to the Nearctic complex of species. All Mexican species of the Neotropical complex were collected in summer (June to August), while the flight period of *Stethantyx nearctica* in Mexico is from October to January. In comparison with Costa Rica, where many species of *Stethantyx* are rather abundant through the year, in Mexico this genus seems to be rather rare because all Mexican species are represented by single or few specimens in available material.

## Supplementary Material

XML Treatment for Stethantyx

XML Treatment for Stethantyx alajuela

XML Treatment for Stethantyx heredia

XML Treatment for Stethantyx mexicana

XML Treatment for Stethantyx nearctica

XML Treatment for Stethantyx osa

XML Treatment for Stethantyx sanjosea
